# The Effect of the COVID-19 Pandemic on School Readiness and Mental Health Concerns: A Prospective Cohort Pilot Study

**DOI:** 10.3390/children13060835

**Published:** 2026-06-20

**Authors:** Christine B. Mirzaian, Tamara Matic, Melissa Lee Wilson, Imani Franklin, Vanessa Castro, Salvador Gonzalez, Seongwook Amos Byun, Alexis Deavenport-Saman, Olga Solomon, Irina Quebles, Marie Kanne Poulsen, Stephanie A. Bughi-Capecci, Larry Yin

**Affiliations:** 1 Department of Pediatrics, Keck School of Medicine of USC, Los Angeles, CA 90027, USA; 2 Children’s Hospital Los Angeles, University Center for Excellence in Developmental Disabilities, Los Angeles, CA 90027, USA; 3 Children’s Hospital Los Angeles, Behavioral Health Institute, Los Angeles, CA 90027, USA; 4 Department of Population and Public Health Sciences, Keck School of Medicine of USC, Los Angeles, CA 90032, USA; 5 San Gabriel/Pomona Regional Center, Pomona, CA 91766, USA

**Keywords:** school readiness, early intervention, COVID-19, autism, mental health

## Abstract

**Background/Objectives**: The COVID-19 pandemic had a negative effect on early intervention (EI) delivery to children with developmental delays or disabilities. This study aimed to compare school readiness of children who received, or attempted to receive, EI before, during, and after the COVID-19 pandemic stay-at-home order. **Methods**: A prospective cohort study was conducted with a single state-funded center that delivers federally mandated EI. School readiness assessments were performed using the Wechsler Preschool and Primary Scale of Intelligence, Fourth Edition (WPPSI-IV). Fisher’s Exact tests, and Kruskal–Wallis ANOVA were performed to compare scores in children who began EI in the three time periods related to the COVID-19 pandemic. **Results**: A total of 56 children were enrolled in this study, the timing of EI start was available in 43, and 22 were able to complete all assessments. Statistically significant differences were found in WPPSI-IV Verbal Comprehension Index T scores (100 ± 15) across the COVID-19 phases, with the lowest scores arising during the pandemic (76.2 ± 9.3); the highest scores, pre-pandemic (98.0 ± 6.2); and intermediate scores, post-pandemic (81.5 ± 10.1, *p* < 0.05). Many children exhibited mental health concerns, with 29/56 (52%) being referred to community-based mental health services. **Conclusions**: In this study, lower scores were reported for markers of school readiness in children who received or attempted to receive EI during the COVID-19 stay-at-home orders compared to those pre- and post-pandemic. High mental health needs were identified, particularly among children with mild–moderate symptoms of autism or those who underwent EI during the COVID-19 stay-at-home orders.

## 1. Introduction

Children who enter kindergarten prepared to learn have a higher probability of fulfilling academic socioemotional milestones [1], which has been linked to a variety of lifetime benefits (e.g., improved well-being, social relations, and economic outcomes) [2,3]. One measure of a child’s growth and development is school readiness. School readiness consists of cognitive, emotional, physical, and social development skills and involves family and community support, including the schools’ ability to meet children’s needs [2]. School readiness is primarily shaped by a child’s environment, and healthy, consistent, and supportive experiences benefit a child’s ability to acquire knowledge and skills and cultivate resiliency [2].

In the spring of 2020, lives were suddenly altered as people worldwide were instructed to social distance and/or stay at home to halt community transmission of the COVID-19 virus [4]. The stay-at-home order, when enforceable, improved COVID-19 incidence and mortality [4], but it also led to interruptions in individuals’ day-to-day routine, family dynamics, and social interactions and connections, including the closing of businesses and schools [5,6]. Children’s experiences at the time of this health crisis were associated with their access to educational opportunities, family and community support, and their age and health during the pandemic [7].

Outcomes of the COVID-19 pandemic show significant influences on children’s mental health, development, and social skills, with availability and access to quality care and education also being affected [7,8,9,10]. Mental health concerns, such as psychological distress, anxiety, depression, and suicidal ideation, increased among the pediatric population, with disproportionate effects reported in underserved communities and communities of color [8,9,10]. Increased educational gaps, decreased wellness visits, growth-related issues, and a rise in adverse childhood experiences (ACEs) have also been reported in the literature [11]. Furthermore, negative effects of the COVID-19 pandemic have been seen in school readiness, as well as case identification and early intervention (EI) delivery for children ages 0 to 5 years with developmental delay or developmental disabilities [7,12]. Early intervention, as mandated by Part C of the Individuals with Disabilities Education Act (IDEA), is designed to improve school readiness by “minimizing the need for special education and related services after infants and toddlers with disabilities reach school age” [13]. However, the long-term impacts on the disruption of EI, including impacts on school readiness, are not yet well understood. This pilot study aimed to compare school readiness of children who had received, or attempted to receive, EI before, during, or after the COVID-19 pandemic stay-at-home order.

## 2. Materials and Methods

A pilot, prospective cohort study was conducted by partnering with a single state-funded center in Southern California, which administers federally mandated EI [13]. The center provided a list of 559 children whose parents had begun or attempted to obtain EI during one of three periods: Phase 1, pre-COVID-19 pandemic (19 March 2019 to 18 March 2020); Phase 2, during the COVID-19 state-mandated stay-at-home order (19 March 2020 to 14 June 2021); and Phase 3, after the COVID-19 stay-at-home order (15 June 2021 to 14 June 2022). The inclusion criteria for this study were children ages 3 to 6 years and their parents who spoke either English, Spanish, or Mandarin. Using the list provided by the center, families were contacted by phone and invited to participate in the study. If they expressed interest, they were provided with additional information about the study and signed consents. They received a $100 gift card for participating. This study was approved by our institution’s Institutional Review Board (IRB).

Four assessments were administered to participants to measure their cognitive, behavioral, emotional, and language development. These assessments included the Wechsler Preschool and Primary Scale of Intelligence, Fourth Edition (WPPSI-IV) [14], the Childhood Autism Rating Scale, Second Edition (CARS-2) [15], the Child Behavior Checklist (CBCL) [16], and the Parenting Stress Index, Fourth Edition, Short Form (PSI-4-SF) [17]. The WPPSI-IV is designed to assess children ages 2 years and 6 months through 7 years and 7 months and can provide information and standardized scores regarding general intelligence. Furthermore, it includes the Full-Scale Intelligence Quotient (FSIQ) and the Verbal Comprehension Index (VCI), as well as the Visual Spatial Index (VSI), Working Memory Index (WMI), and Processing Speed Index (PSI), with standard scores based on a mean of 100 and standard deviation of 15. Given the correlation between general cognitive ability and school readiness [18,19], as well as the specific importance of language ability [19], the WPPSI-IV, FSIQ, and VCI were chosen as primary outcome measures in our analysis as a proxy for school readiness. Since we did not have access to the EI center records for this study, which may have included later diagnoses, such as for autism, we conducted CARS-2 assessments to indicate whether a child had symptoms consistent with autism, as this may have impacted their ability to engage in our assessments, as well as impacted service need and school readiness. The CARS-2 is designed to inform diagnostic hypotheses for children suspected of failing within the autism spectrum disorder (ASD) category [15], and scores indicate minimal to no symptoms of autism, mild to moderate symptoms of autism, and severe symptoms of autism. Although the Autism Diagnostic Observation Schedule, Second Addition (ADOS-2) and the Autism Diagnosis Interview Revised (ADI-R) are regarded as the most traditional, gold standard evaluations of autism [20], the CARS-2 enables the rapid determination of ratings of autism severity and has been comparable to ADOS-2 in previous studies [21]. It was used in this study not to diagnose autism, but to inform us on how to interpret the subjects’ needs and results of additional assessments.

To obtain a more complete picture of the child’s mental health, CBCL was conducted, which is a standardized instrument for the assessment of behavioral and emotional problems in children, including anxiety and depression. The CBCL yields standardized T-scores, with scores of 64 and above generally considered to be clinically significant. Given the significant impact of the COVID-19 pandemic on stress related to parenting [22,23], we administered the PSI-4-SF [17], which has been shown to be an effective and appropriate measure for families, including those from Hispanic/Latino and Spanish-speaking populations [24]. The PSI-4-SF yields scores on subscales including parental distress, parent–child dysfunction, difficult child, and total stress, with higher scores indicating higher stress, and scores >85th percentile considered clinically significant.

The WPPSI-IV and CARS-2 were conducted by a licensed pediatric psychologist, and parents completed the CBCL and PSI-4-SF (in their preferred language, including English, Spanish, or Mandarin), as well as a brief demographic survey.

Our pediatric psychologists generated a report for families based on all scores from the WPSSI-IV, CARS-2, CBCL, and PSI-4-SF. This report was provided to families for their own use with the caveat that it was performed in the context of a research study. However, where appropriate, our psychologist made recommendations and/or referrals to community-based agencies including those for mental and behavioral health, as well as linkage to guidance services and insight into obtaining school-based services. As the EI center has an additional program for children of about 3 years of age with ASD, if information from the study assessments was relevant to the EI center, the results were shared with the family’s permission. The psychologist noted recommendations, and frequency of recommendations were recorded as part of our data collection process.

We compared domains of general cognition and language development, as well as parenting stress and child behavior, between COVID-19 pandemic phases. In addition, we investigated the relationship between ASD severity and mental health and parenting stress. Data analyses were performed in Stata 18 [25], and all variables were presented as mean ± standard deviation for numeric variables and count (frequency) for categorical variables. Skewness and kurtosis were evaluated for all continuous variables, and non-normal distribution was determined (numbers available upon request); thus, nonparametric tests were used for analysis. We tested differences between groups using Fisher’s Exact test for categorical variables and Wilcoxon’s Rank Sum tests (ASD severity) or Kruskal–Wallis ANOVA (COVID-19 wave) for numeric variables. A *p*-value < 0.05 was considered statistically significant. Because this was a pilot study, no a priori power calculations were carried out.

## 3. Results

**Figure 1:**
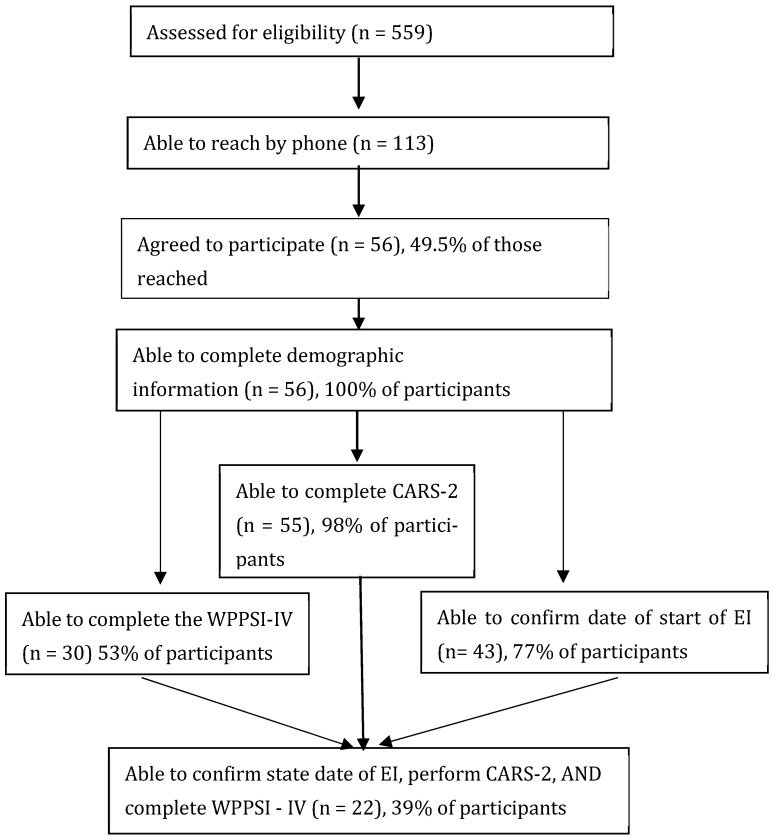
Participant flow diagram.

From the list of 559 families, 113 were reachable by phone and 56 participated in the assessments (participation rate for those able to be reached = 49.5%) (Figure 1). Families who declined to participate shared concerns about time constraints due to work and scheduling, or they were hesitant to have another assessment and/or “doctor’s appointment.”

In total, 39 (69.6%) of the participants were male and 34 (60.7%) were Hispanic/Latino, and the average age of participants was 4.0 ± 1.01 years (Table 1). Timing of EI initiation was available for 43 participants: 7 (16.3%) began EI prior to the pandemic, 19 (44.2%) started during the COVID-19 stay-at-home order, and 17 (39.5%) initiated after the stay-at-home order (Table 1). Data regarding symptoms of ASD (determined by the CARS-2) was available for 55 participants: 32 (58.2%) had minimal to no symptoms of autism, 13 (23.6%) had mild to moderate symptoms of autism, and 10 (18.2%) had severe symptoms of autism.

Among children, there was a statistically significant difference in age according to the pandemic wave. Specifically, pre-pandemic children were older (4.7 ± 1.0) compared to during (4.2 ± 1.0) and after (3.2 ± 0.6) the pandemic (*p* < 0.01, Table 2). No differences were observed across the COVID-19 waves with respect to ethnicity or sex (*p* > 0.05). Among parents, neither sex nor ethnicity differed across COVID-19 waves (*p* > 0.05). In contrast, parental age was significantly different between the COVID-19 waves, with pre-pandemic participants being primarily in the 41–50 age group compared to during and post-pandemic participants who were mostly 31–40 years old (*p* = 0.01).

The WPPSI-IV was administered to all participants; however, 26 (46.4%) children were not able to complete the assessment due to developmental or behavioral challenges. Twenty-two of the children, who completed the WPPSI-IV, were assigned to a COVID-19 phase based on their EI contact record, and they had a CARS-2 score, indicating complete data to be included in the analysis of primary findings.

### 3.1. Primary Findings

Our results in Table 3 show CBCL and PSI-4-SF T-scores were lowest pre-pandemic, highest during the pandemic, and intermediate post-pandemic, although these differences were not statistically significant (*p* > 0.05). There was a statistically significant difference in WPPSI-IV VCI T-scores across the COVID-19 pandemic waves, with the lowest scores found during the pandemic (76.2 ± 9.3), highest scores pre-pandemic (98.0 ± 6.2), and intermediate scores post-pandemic (81.5 ± 10.1, *p* = 0.03). Borderline significant differences were found for the WPPSI-IV FSIQ T-score, with the lowest scores observed during the pandemic (81.9 ± 12.1), highest scores pre-pandemic (102.3 ± 9.7), and intermediate scores post-pandemic (86.4 ± 10.7, *p* = 0.10).

### 3.2. Secondary Findings

Many children in this study exhibited mental health concerns, and 29 out of 56 (51.8%) were referred to community-based mental health services. There were trends in children with mild or moderate symptoms of ASD and in need of mental health referrals. We found that participants with mild or moderate symptoms of ASD had higher referral rates to mental health services (76.9%) than those with no symptoms (48.4%) or severe symptoms of ASD (40.0%), but these differences were not statistically significant (*p* = 0.156). Furthermore, children who underwent EI during the COVID-19 stay-at-home order had higher referral rates for mental health services (66.7%) compared to those pre-pandemic (42.9%) or post-pandemic stay-at-home order (41.2%), but there was no statistical significance (*p* = 0.275). While there were no statistically significant differences in the CBCL, PSI-4-SF, or WPPSI-IV T-scores between ASD severity groups, there was a notable relationship across the domains of the CBCL and PSI-4_SF, with those in the group with minimal to no ASD symptoms having lower scores than those in the group with mild to moderate or severe symptoms (Table 4). Specifically, there was a borderline significant difference in CBCL internalizing scores among those with minimal to no ASD symptoms scoring lower (61.4 ± 12.1) than those with mild to moderate or severe ASD symptoms (67.2 ± 10.7, *p* = 0.09). Higher scores were also observed for those with mild to moderate or severe ASD symptoms (59.1 ± 11.9) compared to those with minimal to no ASD symptoms (53.2 ± 11.5, *p* = 0.07) for the PSI-4-SF difficult child T-score, as well as the PSI-4-SF total stress score, with higher scores observed in the group with mild to moderate or severe ASD symptoms (56.1 ± 11.0) than group with minimal to no ASD symptoms (50.9 ± 10.0, *p* = 0.08).

## 4. Discussion

In this pilot study, we found lower scores on markers of school readiness in children who received or attempted to receive EI during the COVID-19 stay-at-home orders compared to those in the pre- and post-COVID-19 periods. Our results are consistent with those reported by researchers in Cincinnati, Ohio, who conducted a retrospective cohort study that explored Kindergarten Readiness Assessment (KRA) scores pre-COVID-19 pandemic (years 2018 and 2019) and during the COVID-19 pandemic (year 2021) [26]. According to the authors’ findings, mean KRA scores during the COVID-19 pandemic were significantly lower than those in pre-pandemic years, and “lower KRA performance was significantly associated with failing developmental screening after 18 months” [26]. Other studies that explored the impacts of the COVID-19 pandemic also reported decreases or losses in children’s cognitive, motor, and social communication development, including lower use of services, such as developmental therapies and early childhood education [26,27,28]. In addition, our findings are consistent with a recent scoping review evaluating the impacts of the COVID-19 pandemic on elementary students’ social, emotional, and cognitive development [29], finding themes like lack of socialization opportunities, emotional regression, cognitive gaps in learning, and, importantly, inequitable impacts on development. These findings indicate that already marginalized students, including students with disabilities, may have had even greater negative impacts related to the disruption in educational services, including early intervention services [29].

Another outcome of our study is the high mental health needs and referrals to mental health services reported among participants, particularly among children with mild–moderate symptoms of autism or those who underwent EI during the COVID-19 stay-at-home orders. This finding supports the literature reporting that during the COVID-19 pandemic, more than 50% of children with autism experienced a mental health decline, and an increase in behavioral issues was found in the pediatric population with ASD [28,30]. This may be because children with ASD are disproportionately affected by stressful environments and events, particularly related to unexpected changes such as those experienced during the COVID-19 pandemic [28]. In addition, studies on the mental health of children with ASD during the pandemic indicated that parental mental health played an important role in the child’s mental health, and that disruptions in access to educational supports had a significant negative impact [30]. The findings of our study, as well as of similar studies, emphasize the need for service continuity and increased mental health support for children and their families during major events such as the COVID-19 pandemic, which may have important policy implications.

### Limitations

There are a few limitations to this study. First, we did not anticipate a large number of participants with autism. The WPPSI-IV is a test that relies on verbal ability and that requires the participant to sit and engage with the psychologist administering the test for approximately 1 h. This assessment was challenging for many of our participants, especially those with autism; 26 children were unable to complete the WPPSI-IV, and 22 of those were from the group in which we were able to confirm the start date of EI. Only 2 of the children with symptoms of autism were able to complete the WPPSI-IV. This was a major limitation and negatively affected our ability to draw conclusions from this study. This led to a small sample size which was too small to support statistical modeling with covariate adjustment. Thus, for future studies, we recommend using the Leiter International Performance Scale, Third Edition (Leiter-3), a non-verbal test of intelligence, with measures for language, like the Preschool Language Scales, Third Edition (PLS-3), which can be used with children ages 0 to 6 years. Another suggestion is to explore measures of adaptive functioning using the Vineland or Adaptive Behavior Assessment System (ABAS), which includes a domain for communication. In addition, though WPPSI-IV scores are standardized by age group, it is possible that children who were older at the time of the study (i.e., those who were in the pre-pandemic cohort) scored higher related to their age, influencing their abilities and willingness to participate and thereby biasing the results of our study in favor of older children.

The second limitation of this study is that most of the families who agreed to participate expressed concerns about waiting to receive an evaluation for autism or other behavioral concerns for their children. This likely created a selection bias since families with greater concerns were more likely to agree to participate in this study. This gave us important insight into what many families who were continuing to attempt to obtain eligibility or other evaluations through the regional center may benefit from (primarily, referrals to mental health services); however, this decreased the generalizability of our study. Lastly, due to our small sample size, not all associations reached statistical significance, but we were able to see many trends in our data. Our sample size was smaller partly due to the duration of the assessment; the assessment took significantly longer than originally intended, primarily because families had multiple concerns, and extra time was used to engage children with the WPSSI-IV. If the study were to be replicated, it is suggested to budget the time for assessments to 2 h each, rather than 1 h, which is what we estimated in our original study design. In addition, though the original list of eligible families was provided based on the dates of families contacting EI, we were not able to confirm the start date of assistance during a COVID-19 wave for all participants.

Importantly, our study used the WPPSI-IV FSIQ scores and VCI scores as proxies for school readiness. These measures only captured the overall cognitive and language abilities of these children and did not capture additional domains of school readiness, such as social, emotional, and adaptive domains. Thus, our study can provide suggestions for directions of school readiness related to the receipt of EI during phases of the COVID-19 pandemic, but cannot make any definitive conclusions. We hope that our challenges with study design, including the use of the WPPSI-IV, might inform future research examining similar questions and to focus on non-verbal assessments and those including adaptive skills to measure school readiness. In addition, though we had some challenges with data due to our use of a retrospective dataset not originally designed for research, overall, we felt that the academic and community-based partnership was an important collaboration and led to interesting findings and lessons for future studies.

## 5. Conclusions

This pilot study found lower scores on markers of school readiness in children who received or attempted to receive EI during the COVID-19 pandemic compared to pre- and post-COVID-19 phases. In addition, over half of the children who participated in this study presented mental health concerns requiring community-based resources. Many of the families who participated in this study were seeking further support for an autism diagnosis or help to obtain an autism evaluation; however, this concern, in many of these cases, was related to mental health. This suggests that there may be a role for early intervention intake as the first point of contact for families calling for assistance, to be able to provide basic information about community-based mental health resources so that these concerns may be able to be addressed sooner rather than later.

Further studies are needed to evaluate the school trajectory of these children and how additional support can best be provided to make up for the disruptions in learning and developmental care in early childhood.

## Figures and Tables

**Table 1:** General demographic characteristics of the study population.

Child Characteristics		
Age (years) ^1^	N = 56	4.0 ± 1.01 ^1^
Sex **^2^**	N = 56	
Male		39 (69.6%)
Female		17 (30.4%)
Ethnicity **^2^**	N = 56	
Asian		9 (16.1%)
Black or African American		2 (3.6%)
Hispanic/Latino		34 (60.7%)
Other		8 (14.3%)
White		2 (3.6%)
Unreported		1 (1.8%)
COVID-19 Wave (when contacted and initiated EI)		
Pre-COVID-19 Pandemic	N = 43	7 (16.3%)
During COVID-19 Pandemic		19 (44.2%)
Post-COVID-19 Pandemic		17 (39.5%)
Symptoms of autism (by CARS-2)		
Minimal to no symptoms	N = 55	32 (58.2%)
Mild to moderate symptoms		13 (23.6%)
Severe symptoms		10 (18.2%)

^1^ Age (years) is displayed as mean ± standard deviation. ^2^ Frequencies for sex and ethnicity are presented as N (%).

**Table 2:** Demographics and clinical characteristics of the study population stratified by the COVID-19 wave.

Variable ^1^	Pre-COVID-19 Pandemic ^2^ (N = 7)	During COVID-19 Pandemic ^2^ (N = 19)	Post-COVID-19 Pandemic ^2^ (N = 17)	*p*-Value ^3^
Child Characteristics				
Age (years) **^1^**	4.7 ± 1.0	4.2 ± 1.0	3.2 ± 0.6	**<0.01**
Sex				0.45
Female	2 (28.6)	4 (21.1)	7 (41.2)	
Male	5 (71.4)	15 (79.0)	10 (58.8)	
Ethnicity				0.91
Not Hispanic/Latino	3 (42.9)	6 (31.6)	6 (35.3)	
Hispanic/Latino	4 (57.1)	13 (68.4)	11 (64.7)	
Parent Characteristics				
Age				0.01
22–30	1 (14.3)	2 (10.5)	7 (41.2)	
31–40	2 (28.6)	15 (79.0)	9 (52.9)	
41–50	4 (57.1)	1 (5.3)	1 (5.9)	
51–60	0 (0)	1 (5.3)	0 (0)	
Sex				0.37
Female	6 (85.7)	17 (89.5)	17 (100)	
Male	1 (14.3)	2 (10.5)	0 (0)	
Ethnicity				0.83
Not Hispanic/Latino	3 (42.9)	6 (31.6)	5 (29.4)	
Hispanic/Latino	4 (57.1)	13 (68.4)	12 (70.6)	

^1^ Numeric variables are presented as median (IQR), while categorical variables are presented as count (frequency). ^2^ Pre-COVID-19 pandemic (19 March 2019–18 March 2020), during COVID-19 pandemic (19 March 2020–14 June 2021), and post-COVID-19 pandemic (15 June 2021–14 June 2022). ^3^ *p*-values were obtained using Fisher’s exact tests for categorical variables and Kruskal–Wallis ANOVA for continuous variables.

**Table 3:** T-scores for CBCL, PSI-4-SF, and WPPSI-IV according to the COVID-19 wave.

T-Score ^1^	N	Pre-COVID-19 Pandemic ^2^	N	During COVID-19 Pandemic ^2^	N	Post-COVID-19 Pandemic ^2^	*p*-Value ^3^
CBCL	7	58.6 ± 10.1	17	60.4 ± 11.6	17	58.7 ± 7.1	0.90
Depression		59.9 ± 11.8		65.5 ± 15.7		62.6 ± 10.3	0.73
Anxiety		60.3 ± 10.8		64.2 ± 19.0		63.0 ± 9.6	0.36
Anxious/Depressed Internalizing		62.4 ± 11.1		65.8 ± 13.2		63.2 ± 10.7	0.82
Externalizing		52.3 ± 13.3		63.3 ± 14.4		59.4 ± 13.4	0.21
Total		60.4 ± 12.6		67.8 ± 15.9		65.1 ± 11.7	0.41
PSI-4-SF	7		19		17		
Parental Distress		49.4 ± 14.7		53.1 ± 11.3		52.4 ± 11.7	0.65
Parent–Child Dysfunction		51.0 ± 9.3		54.3 ± 9.9		52.5 ± 9.7	0.58
Difficult Child		54.6 ± 12.7		58.3 ± 11.6		55.6 ± 12.8	0.65
Total Stress		51.6 ± 12.6		55.6 ± 11.0		53.8 ± 11.4	0.51
WPPSI-IV							
FSIQ	3	102.3 ± 9.7	11	81.9 ± 12.1	8	86.4 ± 10.7	0.10
VCI	3	98.0 ± 6.2	9	76.2 ± 9.3	8	81.5 ± 10.1	**0.03**

^1^ Presented as mean ± standard deviation. ^2^ Pre-pandemic (19 March 2019–18 March 2020), during Pandemic (19 March 2020–14 June 2021), post-pandemic (15 June 2021–14 June 2022). ^3^ *p*-values were obtained via Kruskal–Wallis ANOVA.

**Table 4:** T-scores for CBCL, PSI-4-SF, and WPPSI-IV according to ASD severity.

T-Score ^1^	N	Overall	N	Minimal to No Symptoms of ASD	N	Mild to Moderate or Severe Symptoms of ASD	*p*-Value ^2^
CBCL	50	63.0 ± 13.7	30	61.2 ± 15.6	20	65.7 ± 10.1	0.28
Depression		62.9 ± 13.1		62.4 ± 12.0		63.8 ± 14.8	0.97
Anxiety		59.3 ± 10.0		58.7 ± 9.3		60.3 ± 11.3	0.87
Anxious/Depressed Internalizing		63.7 ± 11.8		61.4 ± 12.1		67.2 ± 10.7	0.09
Externalizing		59.6 ± 12.0		57.6 ± 14.4		62.7 ± 13.1	0.23
Total		64.8 ± 13.8		62.4 ± 14.3		68.3 ± 12.5	0.15
PSI-4-SF	55		32		23		
Parental Distress		50.7 ± 11.4		48.7 ± 10.4		53.4 ± 12.3	0.16
Parent–Child Dysfunction		52.4 ± 9.3		50.8 ± 9.2		54.6 ± 9.1	0.19
Difficult Child		55.7 ± 12.0		53.2 ± 11.5		59.1 ± 11.9	0.07
Total Stress		53.1 ± 10.7		50.9 ± 10.0		56.1 ± 11.0	0.08
WPPSI-IV							
FSIQ	30	85.7 ± 13.5	28	85.6 ± 12.1	2	87.0 ± 36.8	0.96
VCI	27	82.2 ± 12.5	25	82.6 ± 11.7	2	77.5 ± 26.2	0.81

^1^ Presented as mean ± standard deviation. ^2^ *p*-values were obtained using Kruskal–Wallis ANOVA.

## Data Availability

The data presented in this study are available on request from the corresponding author due to privacy and legal reasons.

## Abbreviations

The following abbreviations are used in this manuscript:

EI	Early Intervention
WPPSI-IV	Wechsler Preschool and Primary Scale of Intelligence, Fourth Edition
CARS-2	Childhood Autism Rating Scale, Second Edition
CBCL	Child Behavior Checklist
PSI-4-SF	Parenting Stress Index, Fourth Edition, Short Form
FSIQ	Full Scales Intelligence Quotient
VCI	Verbal Comprehension Index
ASD	Autism Spectrum Disorder
